# Clinical and economic impact of the introduction of a nucleic acid amplification assay for *Clostridium difficile*

**DOI:** 10.1186/s12941-017-0252-7

**Published:** 2017-12-04

**Authors:** Margaret M. Guinta, Kristen Bunnell, Amanda Harrington, Susan Bleasdale, Larry Danziger, Eric Wenzler

**Affiliations:** 1 College of Pharmacy, University of Illinois at Chicago, 833 South Wood St. Room 164, Chicago, IL USA; 2 Department of Pathology, University of Illinois at Chicago, Chicago, IL USA; 3 Department of Medicine, University of Illinois at Chicago, Chicago, IL USA

**Keywords:** *Clostridium difficile*, Nucleic acid amplification assay, Polymerase chain reaction, Enzyme immunoassay, Outcomes, Costs

## Abstract

**Background:**

The clinical outcomes and cost implications of a diagnostic shift from an EIA- to PCR-based assay for *Clostridium difficile* infection (CDI) have not been completely described in the literature.

**Methods:**

The impact of the PCR-based assay on the incidence and duration of CDI therapy was compared to the EIA assay for patients with a negative CDI diagnostic result. Secondary clinical and economic outcomes were also evaluated. Independent predictors of receipt of antibiotic therapy were assessed via logistic regression.

**Results:**

141 EIA and 140 PCR patients were included. Significantly more patients were started or continued on anti-CDI antibiotic therapy after a known negative assay result in the EIA group (26 patients vs. 8 patients, *P* = 0.002). Duration of antibiotic therapy after a known negative result was significantly shorter in the PCR group (1 vs. 4 days, *P* = 0.029) and a 23% reduction in the number of tests obtained per patient was observed (1.41 ± 0.86 vs. 1.82 ± 1.35*, P* = 0.007). The over fourfold difference in per-test cost of the EIA assay ($8.33 vs. $42.86, *P* < 0.0001) was offset by the overall medication costs required for the increased treatment in the EIA group ($546.60 vs. $188.96, *P* = 0.191). Utilization of the EIA-based CDI assay was associated with increased odds of CDI treatment after a negative test (aOR 4.71, 95% CI 1.93–11.46, *P* = 0.001).

**Conclusion:**

The transition from an EIA to PCR-based assay for diagnosing CDI resulted in a significant decrease in the number of patients treated and the duration of treatment in response to a negative test result. This significant decrease in treatment resulted in decreased costs offsetting the utilization of a more expensive molecular test for patients with a negative CDI diagnostic result.

## Background

*Clostridium difficile* infection (CDI) is the most common cause of infectious nosocomial diarrhea and is responsible for significant morbidity, mortality, and excess healthcare costs [1]. The prevalence of CDI continues to increase in the United States, with the national rate of hospitalizations associated with *C. difficile* tripling from 2001 to 2011 [2]. Given the serious public health burden of CDI, rapid and accurate diagnostic strategies are imperative to reduce disease transmission, optimize antimicrobial therapy, and improve patient outcomes.

Diagnostic laboratory tests for CDI identify either the toxins produced by *C. difficile* or toxigenic *C. difficile* organisms. In clinical practice, methods for diagnosing CDI include nucleic acid amplification tests (NAAT) which detect *C. difficile* toxin genes via polymerase chain reaction (PCR), enzyme immunoassay (EIA) for the *C. difficile* cell wall-associated antigen glutamate dehydrogenase (GDH), or by detection of *C. difficile* free toxins A and B in feces via EIA [3]. Although all of these testing strategies have been employed in different eras and practice settings, toxin A and B detection via EIA was the most commonly employed testing strategy by clinical laboratories in the United States for many years, driven by convenience and decreased labor costs [4]. Unfortunately, the low sensitivity of toxin EIAs relative to toxigenic culture is well-documented and has been reported to be as low as 43.3% by some health systems [3, 5–7]. In practice, multiple EIA toxin tests were often sent in an attempt to overcome the low sensitivity of the test. Furthermore, clinicians may have been more likely to empirically treat and continue treatment for suspected cases of CDI. Given the shortcomings of EIA testing and the improved sensitivity, specificity, decreased labor and turnaround time of NAATs, PCR-based assays have largely replaced EIA methods for diagnosing CDI in clinical practice. Given the high sensitivity and the rapid turnaround time of PCR testing, there is little need for empiric treatment while awaiting the results of the test, treatment despite a negative test result, or for repeat testing during the same symptomatic episode.

In May 2011, the University of Illinois Hospital & Health Sciences System switched from a *C. difficile* A/B toxin EIA (Meridian Premier™, Meridian Bioscience, Cincinnati, OH) to a PCR *tcdB* assay (Cepheid GeneXpert C. difficile, Cepheid, Sunnyvale, CA). The clinical outcomes and cost implications of a diagnostic shift from toxin EIA to PCR for CDI has not been completely described in the literature. The objective of this study was to characterize the antimicrobial use and costs associated with a negative *C. difficile* test diagnosed by two different methods in a single health system. It was our hypothesis that the improved speed and accuracy of the PCR-based assay would reduce the initiation and continuation of anti-CDI antimicrobial therapy in patients with a negative diagnostic assay result, which would in turn reduce costs associated with unnecessary treatment.

## Methods

### Study design

This was a retrospective, single-center cohort study of patients admitted to the University of Illinois Hospital and Health Sciences System, a 495-bed tertiary academic medical center in Chicago, IL, USA. The study was approved by the Office for the Protection of Research Subjects Institutional Review Board with a waiver of consent granted. Patients included in the study were those ≥ 18 years of age who had a negative *C. difficile* diagnostic test (either EIA or PCR) from January 1, 2010 to October 1, 2015. Patients were excluded if they were receiving metronidazole or oral vancomycin for a non-CDI indication at the time of testing, were tested during an outpatient encounter, or if they were being treated for CDI prior to admission. Additionally, patients with a subsequent positive test (EIA or PCR) during the same admission were excluded.

### Data and outcomes

Data obtained via the electronic medical record (EMR) included: age, gender, length of stay, serum creatinine > 1.5 fold premorbid (admission) level, WBC > 15, 000 cells/µL, infectious diseases (ID) consult, Charlson comorbidity index, testing strategy utilized (EIA or PCR), anti-CDI antibiotic therapy (including drug, dose, formulation, frequency, number of doses received), and contact isolation information (initiation and duration of contact isolation). Serum creatinine and WBC were collected within 24 h of the negative test to assess for the presence of severe CDI as defined by the IDSA/SHEA guidelines [5]. During the time the EIA assay was in use, samples were batched and run once daily by the clinical microbiology laboratory while the PCR assay is run on demand. Only patients placed on contact isolation for the documented purpose of presumed *C. difficile* were included in the analysis of contact isolation data. In August 2015, a reflexive contact isolation order was implemented via our EMR associated with the ordering of a CDI diagnostic assay. Prior to this date, orders for contact isolation were entered manually by clinicians. There were no formal hospital-wide educational campaigns directed at prescribers regarding the accuracy of the PCR test or its intended effect on repeat testing or antimicrobial prescribing. Cost information was based on the actual costs accrued by the patient, independent of reimbursement. Hospital costs were calculated by multiplying the average nightly room and board costs by the length of hospital stay, medication costs by multiplying the institution price per dose by the number of antibiotic doses received, and diagnostic tests costs by multiplying the cost per test by the number of tests obtained per patient.

Turnaround time of the assays was calculated as the time between when the test was ordered and when it was resulted in the EMR. To evaluate the impact of assay rapidity, the time to discontinuation of anti-CDI therapy was assessed. This was calculated as the time difference between ordering of the CDI diagnostic assay and discontinuation of anti-CDI antimicrobial therapy. To evaluate the impact of assay accuracy, the number of patients started or continued on anti-CDI antimicrobial therapy after the negative assay result was reported into the EMR was assessed along with the duration of anti-CDI therapy after a known negative result. Other clinical outcomes included the number of tests sent per patient, contact isolation, duration of contact isolation, and length of hospital stay. Economic outcomes included hospital costs, treatment costs, cost of testing strategy, and total overall costs.

### Statistical analysis

Comparisons of categorical data were performed using Chi square or Fisher’s exact test, as appropriate. Comparisons of continuous data were performed using a Student t test or Mann–Whitney U test, as appropriate. A two-tailed significance of < 0.05 was considered statistically significant. Variables associated with CDI treatment despite a known negative test result in the univariate analysis, as evidenced by *P* ≤ 0.2, were included in a logistic regression model using a backwards-stepwise approach to identify significant predictors of receiving CDI treatment after a negative CDI test. Variables were retained in the final model if *P* ≤ 0.05. The Hosmer–Lemeshow test was used to assess goodness of fit. Collinearity was assessed via tolerance and variance inflation factor. All statistical analyses were performed by using SPSS, version 22 (SPSS Inc., Chicago, IL).

## Results

**Fig. 1:**
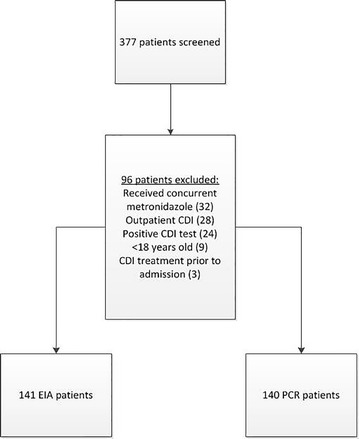
Consort diagram for development of study cohort

During the study period, 377 patients were identified for inclusion. After applying exclusion criteria, 281 patients were included in the final analysis, 141 of whom were tested for CDI via EIA and 140 via PCR (Fig. 1). The majority of patients were excluded due to concurrent non-CDI related metronidazole use, a positive CDI test during the same admission, or the test being performed during an outpatient encounter. Demographics and baseline characteristics were similar between patients tested via EIA or PCR (Table 1). Overall, patients were older in age and had a moderately high baseline severity of illness as indicated by the Charlson Comorbidity Index. Almost half of the patients in both groups would have been classified as having severe CDI based on the IDSA/SHEA guidelines while less than 25% were seen by an infectious diseases specialist.

**Table 1:** Comparison of demographics and baseline characteristics between patients with a negative CDI test diagnosed via EIA or PCR assay

Characteristic	EIA (n = 141)	PCR (n = 140)	*P* value
Age, years	62.8 ± 15.8	59.9 ± 15.8	0.118
Gender, male	63 (44.7)	70 (50)	0.372
Charlson comorbidity index	4.3 ± 2.5	3.8 ± 2.6	0.167
Severe CDI	60 (42.6)	60 (42.9)	0.959
ID consult	22 (15.6)	25 (17.9)	0.613

Data presented as mean ± SD or n (%)

All clinical outcomes assessed were statistically significantly improved in the PCR group compared to the EIA group with the exception of length of stay (Table 2). The median turnaround time of the PCR-based assay was over a day shorter compared to the EIA assay. Median overall time to discontinuation of anti-CDI antibiotic therapy was significantly reduced in the PCR group compared to the EIA group (1 vs. 6 days, *P* = 0.002). Empiric antibiotic therapy was discontinued prior to a known negative CDI diagnostic result in only one patient in each group. There were over three times as many patients started or continued on antibiotic therapy despite a negative test result in the EIA group as compared to the PCR group (26 vs. 8 patients, *P* = 0.002), and those patients started or continued on therapy in the EIA group stayed on therapy significantly longer than patients diagnosed by PCR (4 vs. 1 days, *P* = 0.029). An approximately 23% reduction in the number of CDI tests obtained per patient between the PCR and EIA groups was observed (*P* = 0.007). Importantly, the number of patients placed on contact isolation in the PCR group was over twice that in the EIA group while the duration of contact isolation was reduced by a median of 1 day.

**Table 2:** Clinical and economic outcomes between patients with a negative CDI test diagnosed via EIA or PCR assay

Outcome	EIA (n = 141)	PCR (n = 140)	*P* value
No. of tests sent per patient	1.82 ± 1.35	1.41 ± 0.86	0.007
Turnaround time, hours	32.5 (4.3–91.7)	7.2 (2.4–16.9)	< 0.001
Empiric antibiotic therapy prior to negative result	27 (19.1)	9 (6.4)	0.002
Time to antibiotic discontinuation, days	6 (0–89)	1 (0–14)	0.002
Antibiotic therapy after negative result	26 (18.4)	8 (5.7)	0.002
Duration of therapy after negative result, days	4 (0–88)	1 (0–14)	0.029
Contact isolation	11 (7.8)	24 (17.1)	0.018
Duration of contact isolation, days	2 (1–24)	1 (0–13)	0.008
Length of stay, days	13.5 ± 14.1	14.8 ± 11	0.378
Hospital costs	20,170.21 ± 21,160.43	22,167.86 ± 16,523.10	0.378
Medication costs	546.60 ± 790.39	188.96 ± 179.14	0.191
CDI assay costs	8.33 (8.33–33.32)	42.86 (42.86–171.44)	< 0.0001
Total costs	20,290.00 ± 21,267.75	22,240.31 ± 16,535.50	0.391

Data presented as mean ± SD, median (min–max), or n (%)

^a^Costs presented in 2016 U.S. $

Despite the significantly increased cost of the PCR assay over the EIA assay, there were no significant differences in economic outcomes between the two groups (Table 2). The over fourfold average difference in cost of the CDI test favoring the EIA assay ($8.33 vs. $42.86, *P* < 0.0001) was offset by the medication costs required for the increased treatment of a negative test in the EIA group ($546.60 vs. $188.96, *P* = 0.191). Although a decrease in hospital length of stay was not observed in the PCR group, the average total costs accrued by the patients was cost neutral between the two groups ($20,290.00 vs. $22,240.331, *P* = 0.391).

Candidate variables for the logistic regression model, based on *P* ≤ 0.2 on univariate analysis, included the type of CDI assay used, the number of tests sent, severe CDI, ID consult, and Charlson Comorbidity index. Upon backwards stepwise logistic regression, the type of CDI assay used, severe CDI, and ID consult were retained in the final model. Utilizing an EIA-based CDI assay significantly increased the odds of receiving CDI treatment after a negative test by almost fivefold (aOR 4.71, 95% CI 1.93–11.46, *P* = 0.001) (Table 3). Meeting the criteria for severe CDI also significantly increased the likelihood of receiving treatment (aOR 4.41, 95% CI 1.91–10.19, *P* = 0.001), while obtaining an ID consult significantly decreased the likelihood (aOR 0.31, 95% CI 0.13–0.73, *P* = 0.007). The Hosmer–Lemeshow test indicated a correctly specified model with no evidence of poor fit (*P* = 0.819).

**Table 3:** Independent predictors of receipt of anti-CDI antimicrobial therapy after a negative CDI diagnostic result

Predictor	Univariate model (n = 281)		Multivariate model (n = 281)	
	OR (95% CI)	*P* value	aOR (95% CI)	*P* value
EIA-based CDI assay	3.73 (1.63–8.56)	0.002	4.71 (1.93–11.46)	0.001
Number of tests	1.58 (1.23–2.04)	< 0.001		
Severe CDI criteria met	4.44 (1.99–9.93)	< 0.001	4.41 (1.91–10.19)	0.001
ID consult	0.30 (0.13–0.67)	0.003	0.31 (0.13–0.73)	0.007
Charlson comorbidity index	1.08 (0.94–1.24)	0.260		

*OR* odds ratio, *aOR* adjusted odds ratio

## Discussion

The transition from an EIA- to a PCR-based assay in our institution allowed clinicians to minimize repeat diagnostic tests, to discontinue anti-CDI therapy, and to remove contact isolation precautions more quickly after a negative test result. This is particularly noteworthy as patient isolation has been shown to negatively impact patient comfort and satisfaction and may increase the rate of adverse events [8, 9]. Although the per-test cost of PCR is considerably higher than the EIA assay, the overall strategy in our institution appeared to be cost-neutral for patients with a negative diagnostic test.

Previous studies have assessed the impact of a diagnostic shift to a more sensitive assay for CDI. Grein et al. examined the impact of a shift from EIA, to a two-step testing algorithm, then to exclusive PCR testing in a community hospital and observed a 48% decrease in the number of tests performed per patient and 22% fewer CDI treatment days [10]. This study included active physician education regarding appropriate testing for CDI, which may have inflated the impact of the change in diagnostic test. The impact of the assay on infection control measures was also not assessed and patient outcomes were not individually examined on a granular level and were only reviewed from an aggregate level. Similarly to our study, Catanzaro and Cirone also demonstrated a decrease in the number of patient isolation days, number of tests ordered, and the number of patients continued on therapy despite a negative diagnostic assay with a switch from EIA to PCR-based assay for CDI [11]. The economic impact of the diagnostic shift was not analyzed and patients receiving metronidazole for non-CDI indications were included, likely skewing the true impact of the diagnostic assay on overtreatment. Finally, Peppard and Ledeboer also demonstrated a decrease in the duration of CDI therapy and number of tests sent with a diagnostic shift, although they did not demonstrate a decrease in the duration of contact isolation [12]. Peppard and Ledeboer also did not attempt to assess the severity of symptoms on presentation, which could have accounted for increased antibiotic treatment. After adjustment, we demonstrated the EIA-based assay and severe CDI were associated with a significant increase in the chance of receiving CDI therapy.

Strengths of our study include a real-world testing environment at a time when the prevalence and morbidity and mortality due to CDI are increasing, and a thorough evaluation of both the clinical and economic impacts of a diagnostic assay shift for CDI. Despite the recent debate regarding over diagnosis of CDI with molecular assays [13], we believe our study lends support to this diagnostic method for excluding CDI disease, along with other studies suggesting that molecular assays are more sensitive [14–16]. We did not attempt to evaluate clinical or economic outcomes associated with a positive CDI diagnostic test. Our study adds additional clinical and economic data to existing strictly sensitivity/specificity based evaluations of CDI testing. Limitations of our study include the inherent shortcomings of a retrospective, single-center study design such as small sample size and that we were not able to accurately assess any potential collateral damage resulting from the overuse of metronidazole or oral vancomycin, such as acquisition of vancomycin-resistant *Enterococcus* spp., although this association has not substantiated [17]. Batching of samples during the EIA period could have delayed the time to treatment discontinuation, although we also observed a significantly longer duration of treatment after the negative result was known in this group. The reflexive contact isolation order could have affected the number of patients placed on isolation after August 2015, although the majority of the patients in the PCR group were included between 2011 and 2015. This reflexive order also would not have impacted the decreased duration of contact isolation observed in the PCR group. While the number of tests being sent per patient was significantly decreased after the switch from a toxin EIA to a PCR diagnostic test, patients tested with PCR were still having an average of 1.41 tests sent per admission. Finally, the number of patients initiated on empiric antibiotic therapy while awaiting the results of the CDI assay were low overall. Increased prescriber education regarding the appropriateness of repeat testing following a negative test with a PCR-based method and the importance of empiric therapy if clinical suspicion of CDI is high is warranted within our health system.

## Conclusion

The lack of an optimal testing method for the diagnosis of CDI poses a challenge for clinicians treating this morbid disease. Our study demonstrated clinicians are increasingly confident in the improved timeliness and performance of the PCR-based assay for the diagnosis of CDI and this led to significant improvement in avoidance of therapy in patients with a negative CDI diagnostic test. The transition from an EIA to PCR-based assay for diagnosing CDI resulted in a significant decrease in the initiation and continuation of treatment in response to a negative test result. This significant decrease in treatment resulted in decreased costs offsetting the utilization of a more expensive NAAT for patients with a negative diagnostic assay. Our findings highlight the benefit of a more sensitive diagnostic approach for CDI, findings which are likely to be realized in other healthcare settings.
